# Longitudinal Profiling of IgG and IgA Glycosylation in Pregnancy Reveals Early Associations with BMI, Nulliparity and Conception Mode: The Rotterdam Periconception Cohort

**DOI:** 10.1007/s43032-026-02190-9

**Published:** 2026-09-17

**Authors:** Lotte W. Voskamp, Anna Daniels, Melek Rousian, Mandy van Hoek, Manfred Wuhrer, Régine P. M. Steegers-Theunissen, A. H. Jan Danser, Koen Verdonk

**Affiliations:** 1 Department of Obstetrics & Gynecology, Erasmus University Medical Center, 3015 GD Rotterdam, the Netherlands; 2 Department of Internal Medicine, Erasmus University Medical Center, 3015 GD Rotterdam, the Netherlands; 3 Center for Proteomics and Metabolomics, Leiden University Medical Center, 2333 ZA Leiden, the Netherlands

**Keywords:** Pregnancy, Glycosylation, Immunoglobulins

## Abstract

During early pregnancy, the maternal immune system recalibrates to tolerate the semi-allogeneic fetus. Immunoglobulin (Ig) function is modulated by glycosylation. Prior studies showed increases in anti-inflammatory glycosylation traits (galactosylation and sialylation) in IgG and to a lesser extent in IgA towards the third trimester. This study aimed to characterize IgG and IgA glycosylation patterns throughout pregnancy and assess associations with maternal age, gravidity, body mass index (BMI) and conception mode. In the Rotterdam Periconception Cohort, serum samples from 202 women with singleton pregnancies were collected at 9, 11, 13, 22, and 32 weeks'' gestation. IgG and IgA glycopeptides were analyzed using validated liquid chromatography–mass spectrometry and summarized into galactosylation, sialylation, fucosylation, and bisection traits. Longitudinal changes were assessed with linear mixed-effects models and FDR correction and associations with maternal factors were derived from model estimates. Already in the first trimester, galactosylation and sialylation increased and bisection decreased for both IgG and IgA. IgG galactosylation and sialylation continued rising throughout pregnancy, whereas most IgA traits reversed after the first trimester. Higher BMI was associated with a pro-inflammatory profile (lower IgG galactosylation; higher IgG and IgA fucosylation). Assisted reproduction and nulliparity were associated with anti-inflammatory changes, including higher galactosylation and sialylation. In conclusion, IgG and IgA glycosylation shifts towards an anti-inflammatory profile as early as the first trimester, potentially promoting immune tolerance. Pro-inflammatory changes with higher BMI, and distinct profiles in nulliparous and ART pregnancies, may represent biological pathways linking maternal factors to pregnancy outcomes, supporting glycosylation as a candidate biomarker.

Trial registration number: This study is registered at the Dutch Trial Register (NTR6854).

**Supplementary Information:**

The online version contains supplementary material available at https://doi.org/10.1007/s43032-026-02190-9.

## Introduction

The complex relationship between the maternal immune system and fetal development is fundamental to achieve a successful pregnancy. During pregnancy, the maternal immune system undergoes significant modifications to accommodate and protect the developing fetus, which is genetically distinct from the mother, while also maintaining the maternal protection against pathogens [1, 2]. Most of these modifications occur during the periconceptional period, which spans from three months before conception up to the end of the first trimester of pregnancy [3]. Within this period, critical processes such as implantation, embryogenesis, placentation, and the formation of the maternal–fetal interface are initiated [3]. The maternal immune system plays a crucial role in mediating these processes [2].

**Fig. 1:**
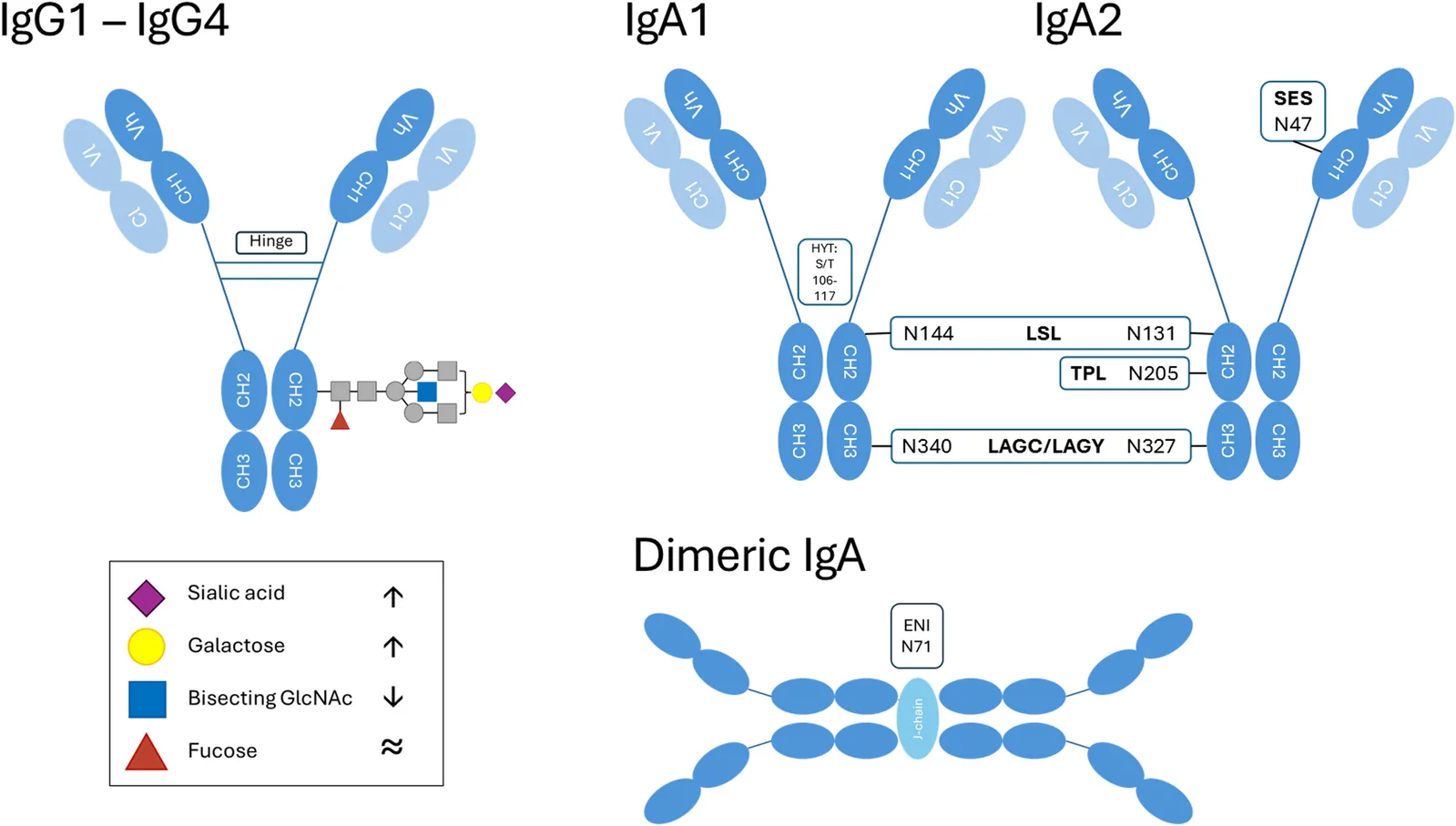
Schematic overview of IgG and IgA subtypes with glycosylation sites and summary of observed pregnancy-associated changes in IgG glycosylation. The analyzed IgA glycopeptides are defined in Supplementary Table 2

Immunoglobulins are important components of the immune system. Of the various classes of immunoglobulins, immunoglobulin G (IgG) is the only one capable of crossing the placental barrier, which makes IgG vital for providing the fetus with passive immunity during pregnancy [4, 5]. Besides IgG, immunoglobulin A (IgA) is the second most abundant immunoglobulin in serum and plays a key role in non-inflammatory immune defense and pathogen neutralization, yet its function in pregnancy remains largely unexplored [6, 7]. Both IgG and IgA are composed of two heavy and two light chains, and their function is modulated by glycosylation, the process by which carbohydrate chains (glycans) are enzymatically attached to proteins [8]. The attached glycans can be modified through galactosylation (addition of galactose), sialylation (addition of sialic acid to galactose), fucosylation (addition of a fucose), and bisection (addition of a bisecting N-acetylglucosamine, contributing to the overall glycosylation profile (Fig. 1) [8]. A recent comprehensive review summarized the diverse roles of glycosylation in human reproduction, including trophoblast invasion, placental development, and maternal–fetal immune regulation [9]. Notably, immunoglobulin glycosylation was highlighted as an important component of maternal immune adaptation, as it influences antibody structure and function, particulary by modulating their affinity for Fc receptors [8–11]. In general, increased galactosylation and sialylation are associated with anti-inflammatory activity, while increased bisection may have pro-inflammatory effects [8, 11–13]. IgG and IgA glycosylation profiles are affected by different conditions, including a more pro-inflammatory pattern observed with aging, obesity, diabetes or auto-immune disease [10, 11, 14, 15].

Previous studies observed that in uncomplicated pregnancies, galactosylation and sialylation of Fc glycans of IgG increase from the first trimester to the second or third trimester of pregnancy [16, 17]. This higher galactosylation is also observed in neonatal cord blood IgG measured at birth, even compared with maternal blood, possibly as a result of selective transport across the placenta [18]. In contrast, changes in serum IgA glycosylation during pregnancy have been examined in only a few studies, highlighting a significant gap in current knowledge [19–21]. Moreover, both IgG and IgA glycosylation patterns have not yet been studied within the periconceptional period. We hypothesized that pregnancy induces specific shifts in antibody glycosylation early in gestation, reflecting a transition toward a more complex, anti-inflammatory profile, including increases in galactosylation and sialylation and decreases in bisection and fucosylation. These changes may be influenced by maternal and pregnancy-related factors known to affect immune adaptation. For example, nulliparous women have a higher risk of immune-mediated complications such as pre-eclampsia and may exhibit a more pro-inflammatory profile [22]. Assisted reproductive technologies (ART) are used in cases of subfertility and involve exogenous administration of estrogen and progesteron, which may influence immune adaptation [23].

The primary objective of this study was to characterize both IgG and IgA glycosylation profiles throughout pregnancy, with a focus on the first trimester, and in cord blood. As a secondary objective, we assessed associations with maternal age, body-mass index, conception mode and parity.

## Methods

### Study Design

This study used data from the VIRTUAL Placenta study, a subcohort of the Rotterdam Periconception Cohort (Predict study), an ongoing longitudinal prospective cohort study that has been including women periconceptionally between 2017 and 2018 at the Erasmus University Medical Center, Rotterdam, the Netherlands [24, 25]. Eligibility criteria for inclusion in the Predict study were women with a singleton pregnancy above 18 years old, with a gestational age (GA) of less than ten weeks. Pregnancies ending in miscarriage or stillbirth were excluded due to incomplete follow-up, while perinatal deaths were excluded to avoid bias from severe complications.

### Data Collection

At inclusion, participants were asked to complete a questionnaire on their general health and medical and obstetrical history. At least two study visits were scheduled in the first trimester at 7-, 9-, 11- and 13 weeks GA. During the intake, weight and height were measured to calculate body mass index (BMI). For natural pregnancies with regular cycles between 25 and 35 days, GA was calculated from the first day of the last menstrual period (LMP). If the LMP was unknown or cycles were irregular, GA was determined using Crown-Rump-Length (CRL) as this is more accurate [26]. If there was a discrepancy of more than six days between the two methods, the GA based on CRL was considered accurate. For pregnancies conceived through in vitro fertilization (IVF) with or without intra-cytoplasmic sperm injection (ICSI), GA was calculated from the day of oocyte retrieval plus fourteen days. In cases of cryopreserved embryo transfer, GA was by adding 17 or 19 days to the embryo transfer date for day 3 and day 5 embryos, respectively.

### Glycosylation Measurements

Venous serum samples were collected during study visits at approximately 9, 11, 13, 22, and 32 weeks GA and directly transported to the laboratory. Umbilical cord blood samples were obtained directly at the time of delivery. For participants who gave birth outside the study center, including deliveries at affiliated hospitals or at home, samples were collected in vacutainer tubes, maintained at room temperature, and dispatched by post to the laboratory within 48 h of collection.

Blood samples were allowed to clot for at least 60 min, and centrifuged at 2000 g for 10 min at 4 °C. Serum was aliquoted (4 × 0.5 ml) and immediately stored at − 80 °C. IgG and IgA glycosylation analysis was performed at the glycopeptide level following a previously validated protocol [27]. Briefly, IgG and IgA were affinity-captured, subjected to reduction, alkylation, and proteolytic cleavage with trypsin, followed by nano-scale LC–MS analysis. Data were processed using the proprietary software LaCyTools, as described previously [28]. Peptide- and site-specific glycosylation was normalized to 1.0 for the sum of all glycosylation variants of a specific peptide. For each of the peptides, glycosylation traits were calculated reflecting specific glycosylation motifs (see Supplementary Table 1). Laboratory personnel conducting LC–MS analysis were blinded to participant clinical characteristics. An overview of IgA glycopeptide clusters, corresponding IgA subclasses, and glycosylation sites indicated by residue number is presented in Supplementary Table 2 [17, 29].

## Statistical Analysis

### Descriptive Analysis

Baseline characteristics were reported as mean and standard deviations, median and interquartile range for non-normally distributed variables, or number and percentage for categorical variables. To assess relationships between glycosylation traits, we calculated pairwise correlation coefficients using the Spearman method for each trimester separately.

### Primary Analysis

For all glycosylation traits, we calculated the mean and standard deviation at each time point. To assess changes between time points, linear mixed-effects models were fitted for each trait, with timepoint as a fixed effect and a random intercept for each subject to account for repeated measurements. Estimated marginal means were compared using pairwise t-tests between predefined time points, and p-values were adjusted for multiple comparisons using the False Discovery Rate (FDR) for the outcomes [30]. P-values < 0.05 were considered statistically significant. For reporting, figures displaying estimated glycosylation trajectories across time points were generated for each group. For binary variables (e.g., presence/absence of complications), the estimated trajectories were plotted for each group, facilitating a visual comparison across different pregnancy outcomes. For Body Mass Index (BMI), three categories were used to facilitate the interpretation of trajectories across different BMI cut-offs: underweight (< 18.5), normal weight (18.5–25), overweight (25–30), and obesity (≥ 30).

### Secondary Analyses

Linear mixed‑effects regression models were used to examine associations between maternal and pregnancy characteristics (including age and BMI at conception, nulliparity, and mode of conception) as independent variables and glycosylation traits as dependent outcomes. Nulliparity was defined as no prior pregnancies beyond 16 weeks gestational age. Mode of conception was classified as natural if pregnancy occurred spontaneously or with the aid of ovulation induction or intrauterine insemination. Assisted reproductive technology (ART) included pregnancies conceived IVF or ICSI. For each model, we tested for significant interactions between GA and the independent variable. Models including pregnancy characteristics were adjusted for maternal age and BMI, with BMI interactions included where applicable. Cord blood IgG and IgA measurements were standardized to Z-scores prior to analysis, and associations between glycosylation traits and maternal characteristics were examined using Spearman rank correlation, reporting correlation coefficients (r) and corresponding p-values.

### Post-hoc Analyses

We performed a post-hoc sensitivity analyses to explore whether the associations observed for nulliparity were confounded by mode of conception (spontaneous vs. IVF/ICSI). The analysis was limited to women in their first pregnancy, and pregnancies conceived via ovulation induction or intrauterine insemination were excluded due to limited numbers and heterogeneous treatment protocols. Statistical analyses were performed using R (version 4.3.2).

### Ethical Approval

Informed consent was obtained from all participants in accordance with the Declaration of Helsinki, and this study was approved by the local ethics committee (MEC-2004–227).

## Results

### Study Population and Baseline Maternal Characteristics

A total of 202 ongoing pregnancies were included in the current study. Baseline characteristics are depicted in Table 1. Mean age at conception was 32 years, and median BMI was 25.0 kg/m^2^. Nulliparous women (n = 113) represented 55.9% of the cohort, and 83 pregnancies (41.5%) were conceived through IVF or ICSI. The majority of women (n = 156) had glycosylation measurements available for four gestational time points. A smaller number had five (n = 4), three (n = 34), two (n = 7), or one measurement (n = 1).

**Table 1:** Maternal demographic and pregnancy characteristics

	N = 202	N missing
Patient characteristics		
Maternal age, mean (SD)	32.1 (4.4)	
Maternal BMI, median [IQR]	25.0 [22.2, 28.8]	
Country of origin, n (%)		6 (3.0)
Dutch	170 (84.2)	
Western, not Dutch	6 (3.0)	
Non-Western	20 (9.9)	
Nulliparity, n (%)	113 (55.9)	1 (0.5)
Smoking periconceptional, n (%)	25 (12.3)	9 (4.5)
Pre-existing hypertension, n (%)	5 (2.5)	2 (1.0)
Pre-existing diabetes mellitus, n (%)		
Type I	3 (1.5)	
Type II	0 (0.0)	
Pregnancy characteristics		
Mode of conception, n (%)		
Spontaneous	103 (51.0)	
Ovulation induction	9 (4.5)	
Intra-uterine insemination	7 (3.5)	
IVF	33 (16.3)	
ICSI	50 (24.8)	
Gestational age at birth in days, median [IQR]	273 [265, 278]	
Neonatal birthweight percentile, median [IQR]	46 [21, 72]	
Pregnancy complication, n (%)		2 (1.0)
Preeclampsia	7 (3.5)	
with onset < 34 weeks	3 (1.5)	
Pregnancy induced hypertension	15 (7.4)	
Gestational diabetes	31 (15.3)	

### Correlation of Glycosylation Traits

To investigate the relationships between different glycosylation traits, we computed correlation matrices for each trimester. The analysis revealed several key patterns, primarily defined by the type of glycan modification (such as O- versus N-glycosylation, bisection, galactosylation), antibody (sub-)class and glycosylation site. Overall, correlations between glycosylation traits were found to be comparable between the three trimesters (Supplementary Figures S1-S3). Detailed results of correlation analyses can be found in the Supplementary Data.

### Changes in Glycosylation Across Pregnancy

#### N-glycosylation of IgG

**Fig. 2:**
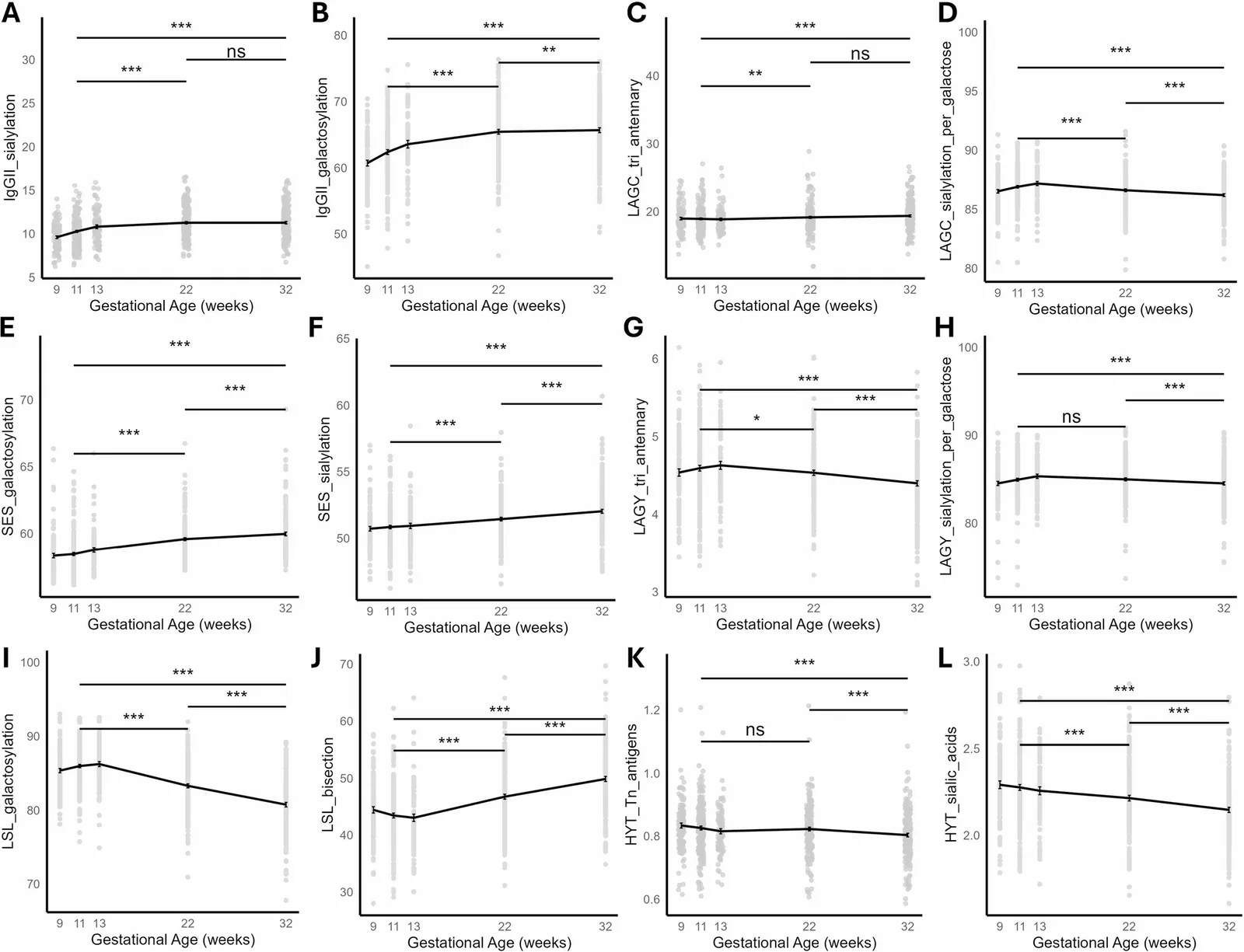
Relative abundance of glycosylation (selection)

For all subtypes of IgG, we observed a significant increase in galactosylation as well as sialylation within the first and second trimesters. Notably, sialylation showed a more prominent increase than galactosylation, leading to an increase in sialyation per galactose. This effect was strongest for IgG2 (Fig. 2 A, B).

In contrast, bisection significantly decreased in IgG1during the first trimester, from 15.88 (± 2.86) at week 9 to 15.13 (± 2.58) at week 11 (p = 0.008), and further to 14.79 (± 2.63) at week 13 (p = 0.019). Bisection in IgG2 and IgG4 showed slight, but non-significant declines. After 13 weeks, bisection levels in all IgG subclasses increased.

At 11 weeks, the mean percentage of fucosylation was 91.8% for IgG1and 99.3% for IgG2 and this remained unchanged during the full course of gestation. All measured means of glycosylation traits are reported in Supplementary Table 3 and depicted in Supplementary Figure S4.

### N-glycosylation of IgA

Different patterns were observed for IgA glycosylation traits. For LSL, TPL, LAGC and LAGY, bisection showed a decreasing trend between 9 and 13 weeks gestation, while sialylation and galactosylation increased. After 13 weeks, the directions change, with a significant increase in bisection and decrease in galactosylation and sialylation towards the second and third trimester for LSL, TPL and LAGY (Fig. 2 I and J, Supplementary Table 3, Supplementary Figure S4). In contrast, galactosylation in LAGC remained stable after the first trimester (99.34%−99.36%).

On both LAGC and LAGY sites, sialylation per galactose increases during the first trimester, and then decreases towards the third (Fig. 2 D, H). However, while tri-antennary LAGC increased between the first and third trimester (18.91 to 19.35, p < 0.001), tri-antennary LAGY decreased (4.63 to 4.40, p < 0.001) (Fig. 2 C, G).

In contrast to the IgA glycosylation sites described above, bisection at the SES site remained stable in the first trimester and slightly decreased towards the third trimester. SES fucosylation increased only between the first and second trimester, while both galactosylation and sialylation showed a continuous increase across all trimesters (Fig. 2 E, F, Supplementary Table 3).

At the monitored glycosylation site of the joining chain (ENI), sialylation significantly decreased between the first, second, and third trimesters, while the abundance of hybrid-type glycan structures increased.

### O-Glycosylation of IgA (HYT region)

Tn antigens showed a slight decrease over pregnancy, from 0.83 relative abundance at week 9 to 0.80 at week 32, with a significant decline between the second and third trimester (p < 0.001, Fig. 2 K). Galactoses of the HYT hinge region glycopeptide slightly increased in pregnancy, from 3.66 at 11 weeks to 3.69 at 32 weeks (*p* < 0.001), as did GalNAc levels and galactoses per GalNAc. Sialylation in the HYT region gradually decreased throughout pregnancy, as indicated by a decline in total sialic acids (2.29 at week 9 to 2.15 at week 32), disialylated O-antigens (3.05 to 2.75), and sialic acids per galactose (0.63 to 0.58).

### Associations with Clinical Parameters

**Fig. 3:**
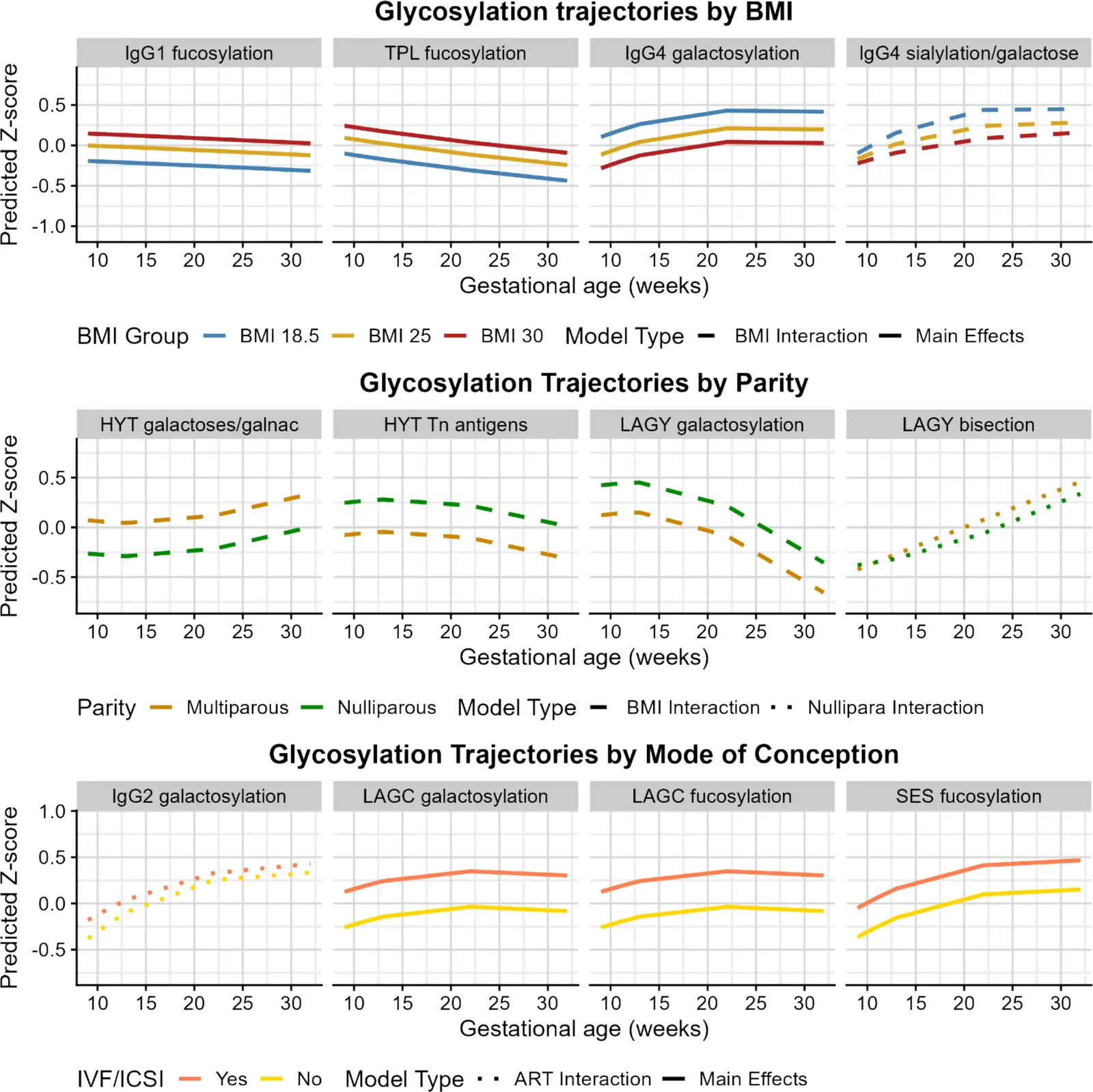
Trajectories of glycosylation traits based on linear mixed regression models. Differences for body-mass index (BMI), nulliparity, assisted reproduction technologies (ART)

Table 2 presents the significant main associations between the exposure variables and IgG glycosylation as derived from the linear mixed regression models. Only variables that showed a significant overall difference throughout pregnancy (main effect) are included in this summary. For some glycosylation traits, an interaction effect with GA was observed, indicating differences in trajectory over pregnancy rather than a consistent increase or decrease. These interactions are not included in the table but are described in detail in the text and visualized in Fig. 3.

**Table 2:** Significant associations between maternal characteristics and IgG/IgA glycosylation across pregnancy

Variable	Age ^(1)^	BMI ^(2)^	Nulliparity ^(3)^	IVF/ICSI pregnancy ^(3)^
IgG1	x	Fucosylation ↑	X	Galactosylation ↑ Sialylation ↑ Hybrid ↑
IgG2	x	X	X	x
IgG4	x	Galactosylation ↓ Sialyation ↓	X	X
LAGC	x	X	X	Fucosylation ↑ Galactosylation ↑
LAGY	x	X	Galactosylation ↑	X
LSL	x	X	X	X
TPL	x	Fucosylation ↑	X	Sialylation ↑ Sialy/Galactosylation ↑
SES	x	Sialylation ↑ Tri-antennary ↑	X	Fucosylation ↑ Sialylation ↑ Tri-antennary ↑
ENI	x	Fucosylation ↑	X	X
HYT	x	Galactoses ↓ Galactoses per GalNac ↑ Tn antigens ↓	Galactoses ↓ Galactoses per GalNAc ↓ Tn antigens ↑	X

^(1)^ Adjusted for maternal BMI; ^(2)^ Adjusted for maternal age; ^(3)^ Adjusted for maternal age and BMI. X: no significant main effect of exposure on glycosylation

### Age

In a crude model, maternal age was associated with lower fucosylation at IgG1 and ENI sites, and increased bisection at IgG4 during the first half of pregnancy. After adjusting for body mass index, no significant associations were observed for any of the investigated glycosylation traits with maternal age at conception.

### Body Mass Index

Women with a higher BMI exhibited significant differences in glycosylation patterns across both IgG and IgA. For IgG, differences were observed in the IgG4 subclass, where women with a higher BMI have significant lower levels of galactosylation and sialylation throughout pregnancy. Additionally, the IgG4 sialylation per galactose increases less in women with a higher BMI (Fig. 3). Women with a high BMI showed increased core fucosylation of IgG1, as well as at IgA N-glycosylation sites TPL and the joining chain ENI. Moreover, sialylation and sialylation per galactoses were slightly higher in the first trimester, but comparable at 22 and 32 weeks. In the SES region, higher sialylation and tri-antennary structures were observed for women with higher BMI. In the HYT region, lower levels of galactoses and Tn antigens were observed, alongside higher levels of galactoses per GalNAc in women with higher BMI (Figure S5).

### Nulliparity

Nulliparous women exhibited different glycosylation patterns compared to multiparous women. In the HYT region, nulliparity was associated with significant lower levels of galactose and galactose per GalNAc, alongside higher levels of Tn antigens (Fig. 3). At N-glycosylation sites, a trend with higher galactosylation and sialylation was observed for nulliparous women (Figure S6)**.** However, this was only statistically significant for IgA site LAGY. At that same side, there was a significant different trajectory for nulliparous women, with an attenuated increase in bisection throughout pregnancy (Fig. 3).

### Mode of Conception

We compared glycosylation patterns between pregnancies conceived spontaneously and with ART. Galactosylation levels were significantly higher in IgG1, IgG2, and LAGC. In IgG2, the trajectory differed by conception mode, with ART pregnancies showing higher levels in the first trimester, followed by a less steep increase thereafter (Fig. 3). Sialylation was increased in IgG2, LAGC, SES, and TPL, and fucosylation was increased in LAGC and SES. Moreover, in the SES region, tri-antennary structures increased in both groups, but ART pregnancies consistently exhibited higher levels throughout gestation (Figure S7).

### Sensitivity Analysis

Since nulliparity was more common in the ART group than in spontaneous pregnancies (77.1% vs. 35.9%), and both ART and nulliparity were individually associated with differences in glycosylation patterns, we conducted a sensitivity analysis to disentangle potential overlapping effects. In this analysis, we stratified by mode of conception (spontaneous vs. IVF/ICSI), focusing on the effect of nulliparity. Ovulation induction and intrauterine insemination were excluded due to small sample sizes and differing protocols. Details on baseline characteristics for this sensitivity analysis are depicted in Supplementary Table 5 and results of the linear regression models in Supplementary Table 6 and Supplementary Figure S8.

Overall, the direction of effects remained consistent, although differences in statistical significance emerged across subgroups. Notably, the glycosylation differences observed at IgA site LAGY in nulliparous women were replicated exclusively within the IVF/ICSI subgroup. In contrast, the decreases in HYT region galactoses and galactoses per GalNAc, as well as the increase in Tn antigens in nulliparous women, were no longer statistically significant following stratification, likely reflecting reduced statistical power within the subgroups. When analyses were restricted to spontaneously conceived pregnancies, LAGC and TPL bisection and sialylation per galactose were significantly higher in nulliparous women (Figure S8).

### Neonatal Cord Blood

**Fig. 4:**
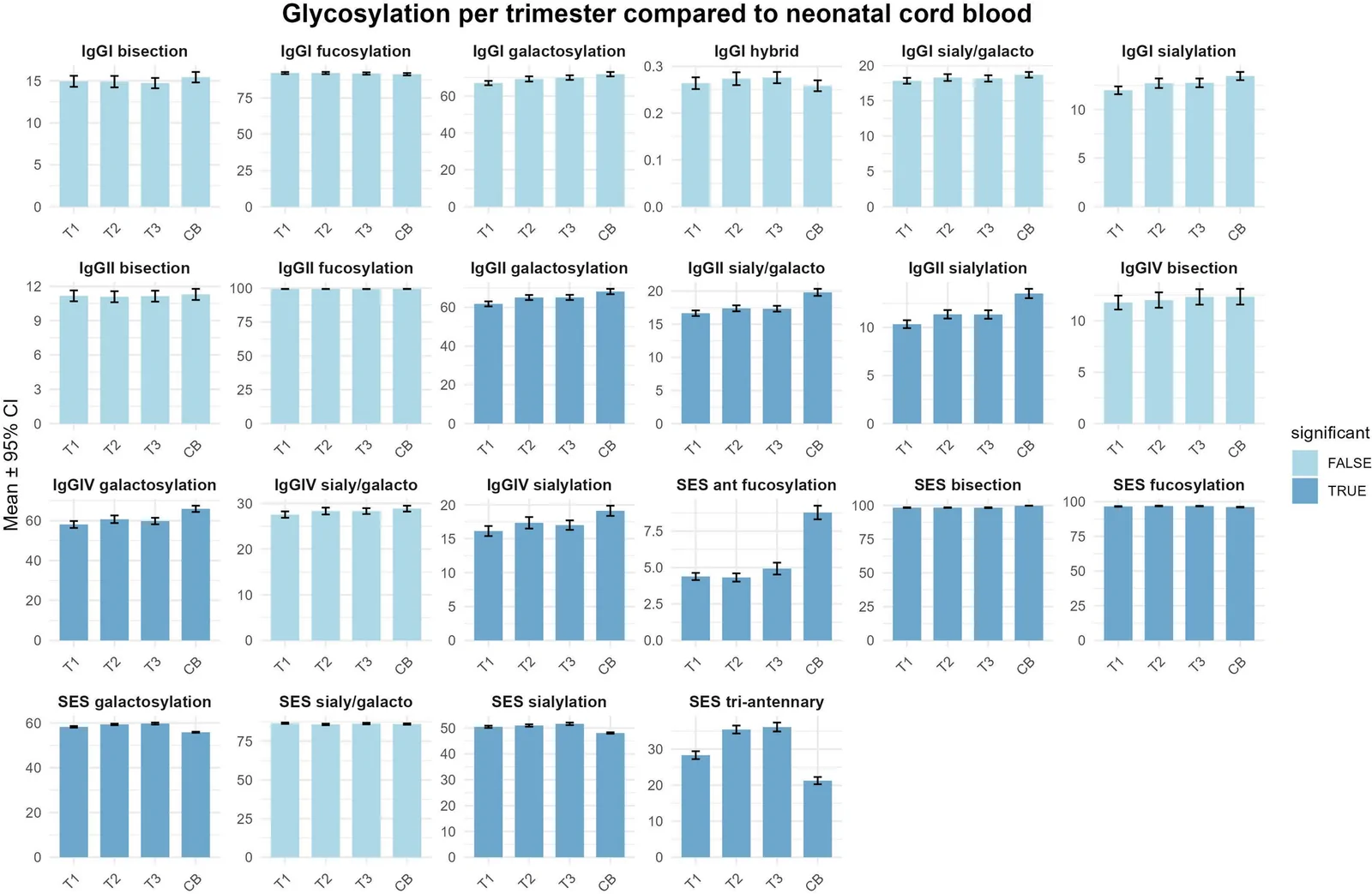
Means and 95% confidence intervals of glycosylation traits measured in neonatal cord blood after delivery (CB) compared to measurements in maternal blood in the first, second, and third trimester. *CI* confidence interval; *sialy/galacto* sialylation per galactose; *SES* *ant fucosylation* SES antennary fucosylation

Cord blood samples were available for 78 neonates. The median GA at delivery was 271 days (range 212–294). IgG and SES glycosylation traits could be measured and their mean values with 95% confidence intervals over the three trimesters and in cord blood are depicted in Fig. 4 and Supplementary Table 4. For IgG glycosylation traits, both galactosylation and sialylation were significantly higher in CB compared to the third trimester in subtypes IgG2 and IgG4, and a similar trend was observed for subtypes IgG1. Only for IgG2, sialylation per galactose was significantly higher. For SES, galactosylation, sialylation, bisection, and fucosylation were all significantly lower in cord blood compared to maternal blood in the third trimester. In contrast, fucosylation of SES antennary glycans was higher. Other IgA glycosylation traits could not be detected in cord blood samples.

### Correlations with Clinical Outcomes

Among maternal characteristics, a higher BMI was weakly associated with increased fucosylation of IgG1 and IgG2 (r = 0.27 and 0.22, respectively) as well as IgG2 sialylation and sialylation per galactose (r = 0.24 and r = 0.23). No associations with an r > 0.20 were observed for ART pregnancies. For nulliparity, a negative association was found only between IgG1 hybrid (r = −0.29) and SES bisection (r = −0.21) in cord blood. Antibody glycosylation was further evaluated in relation to neonatal outcomes. GA at birth was positively correlated with sialylation, galactosylation, and tri-antennary glycans in the SES region, likely reflecting increased post-translational modification of IgA, which is produced by the fetus mainly in the late third trimester. Some sex differences between male and female fetuses were observed, e.g., male fetal sex was weakly correlated with lower IgG4 bisection (r = 0.21) and higher IgG1 hybrid glycans (r = 0.25). Moreover, a higher birthweight percentile correlated with higher abundance of IgG2 sialylated and IgG1 hybrid glycans (r = 0.23 and 0.24, respectively). All correlations between glycosylation patterns in cord blood and clinical parameters are depicted in Supplementary Figure S9.

## Discussion

### Principle Findings

In this cohort of 202 pregnant women, we observed that immunoglobulin glycosylation undergoes significant changes already within the first trimester. For IgG, galactosylation and sialylation increased throughout pregnancy. Bisection decreased between week 9 and 13 for IgG1 and IgG2, but then increased toward the third trimester in IgG1 and IgG4. For most IgA N-glycosylation sites (LSL, TPL, LAGY and LAGC), we observed a distinct switching point at the end of the first trimester. Between 9 and 13 weeks, galactosylation and sialylation tended to increase while bisection decreased, whereas from 13 to 32 weeks, these trends reversed.

Pregnancies conceived through IVF/ICSI generally exhibited higher levels of galactosylation, sialylation, and fucosylation on both IgG and IgA glycosylation sites. Moreover, women pregnant for the first time displayed reduced galactosylation of the O-glycans (HYT), as well as increased sialylation in IgG4, TPL, and LAGY sites during the first trimester. Glycosylation traits in neonatal cord blood showed higher levels of IgG galactosylation and sialylation compared to maternal third trimester serum, and weak correlations with maternal smoking and birthweight.

### Comparison to Current Understanding in the Field

Several studies have investigated changes in IgG N-glycosylation in pregnancy, and observed comparable patterns, including the increase of galactosylation and sialylation, and a decrease of bisection between the first and third trimester [16, 17]. Generally, an increase of galactosylation and sialylation reflects an anti-inflammatory, tolerogenic environment [12, 13]. Previous research showed that it leads to more complement dependent cytotoxicity (CDC) outside of pregnancy [12]. Pregnancy is known to increase activity of the complement system [31], and this could be mediated by glycosylation changes of IgG1. Functionally, an increase in sialylation may reduce binding to activating Fcγ-receptors, thereby dampening the inflammatory response. Likewise, DC-SIGN has been suggested to convey anti-inflammatory properties of IgG sialylation [32].

Interestingly, we observed that bisecting GlcNAc levels changed dynamically throughout pregnancy, with a significant decrease during the first trimester followed by an increase after week 13. While bisection may to some extent influence immune effector functions, its mechanistic role in endogenous IgG remains poorly defined. In the production of therapeutic antibodies, bisecting GlcNAc can enhance antibody-dependent cellular cytotoxicity (ADCC) by interfering with core fucosylation, thereby increasing FcγRIIIa binding and promoting a pro-inflammatory response [13, 33, 34]. However, evidence for a similar inverse relationship between bisection and fucosylation in endogenous antibodies is limited, and this mechanism may not translate to physiological settings. Still, changes in bisection have been associated with autoimmune and inflammatory conditions, suggesting a broader immunomodulatory role [35].

Together, high galactosylation and sialylation together with low bisection in early pregnancy may thus contribute to maternal immune tolerance. In later gestation, the gradual rise in bisection may reflect immune recalibration, enhancing maternal defense against infections while preserving fetal tolerance [36]. This immune shift may also serve as a preparation for labor, which is recognized as a coordinated inflammatory process involving cytokine signaling and immune cell activation [37].

Regarding IgA, however, the functional implications of its glycosylation remain underexplored, particularly in relation to effector functions. Similar to a previous smaller cohort study (n = 29), we observed lowered IgA LAGY and LSL bisection levels in the first trimester, with an increase later in pregnancy. However, by zooming in on the first trimester, we observed that this is preceeded by a decrease between 9–13 weeks of gestation, as in IgG1 bisection. Moreover, while we observed a similar increase for LAGC tri-antennary glycans between the first and third trimester, these decreased at the LAGY site. Another key difference was that no significant changes in fucosylation were observed at either site. These differences underscore the added value of our study in expanding the limited knowledge on IgA glycosylation during pregnancy, particularly by capturing dynamic changes already occurring within the first trimester.

Furthermore, regarding IgA1 hinge region O-glycosylation we found a decrease of sialic acids per galactose. In combination with a decrease in Tn antigens and an increase of galactoses per GalNAc, these findings suggest a general shift from immature towards more mature, galactosylated structures over the course of pregnancy, thereby supporting the anti-inflammatory state.

### Maternal and Pregnancy Characteristics

The absence of significant associations between maternal age and glycosylation changes may be explained by the narrow age range in our cohort (IQR 28–35 years). Previous studies have shown that IgG glycosylation tends to be more pro-inflammatory at younger and older age extremes, while remaining relatively stable around the early thirties [15].

Outside of pregnancy, higher BMI correlates with reduced IgG galactosylation, reflecting a more pro-inflammatory state [38]. We indeed observed lower galactosylation and sialylation in subtype IgG4, though not for IgG1 or IgG2. In contrast, a recent study found higher A2G2S1 levels in third-trimester IgG from obese women compared to those with normal weight, but did not distinguish between IgG subtypes and within a smaller sample size [39]. Our observation of increased fucosylation for IgG1, but not for IgG2, in women with a higher BMI aligns with studies outside of pregnancy [40]. To date, the associations between BMI and IgA N- or O-glycosylation have not been extensively investigated, and no relevant literature on this topic has been identified.

In pregnancies conceived through ART, we observed a generalized increase in glycan complexity, particularly in galactosylation, sialylation, and fucosylation across multiple IgG and IgA sites, reflecting a more anti-inflammatory glycan profile. Differences between naturally conceived pregnancies and those achieved through assisted reproductive technologies may be influenced by confounding factors such as underlying comorbidities causing subfertility. Additionally, the hormonal therapies used in fertility treatments could impact glycosylation patterns, contributing to the observed variations. For example, estrogen has been identified as a modulator of IgG glycosylation. A previous study showed that estradiol promotes anti-inflammatory IgG glycosylation patterns, increasing galactosylation and sialylation while reducing bisection [41]. Fucosylation appeared unaffected by estradiol [41]. For IgA glycosylation, there is no current literature on the influence of estrogen or progesterone.

Women who were pregnant for the first time had lower levels of galactose and galactose per GalNAc, alongside higher levels of Tn antigens in O-glycosylated hinge region of IgA1 compared to those who had been pregnant before. A previous study by Bondt. et al. (2016) showed that there was a slight increase in galactosylation of this region that persisted postpartum, which would explain the lower galactosylation in women that have not been pregnant before [19]. However, in the sensitivity analysis stratified by conception mode, this associations was only seen in IVF/ICSI pregnancies. This could potentially reflect the impact of assisted reproduction technologies, or limited statistical power in the spontaneous pregnancy group. Additionally, we observed a more anti-inflammatory N-glycosylation profile in women who were pregnant for the first time, a finding not previously described.

### Neonatal Cord Blood

Previous studies have primarily focused on IgG placental transfer, demonstrating that galactosylated and sialylated Fc glycoforms are preferentially enriched, possibly due to selective binding to the neonatal Fc receptor [18]. Consistent with these findings, our analysis revealed significantly higher levels of IgG galactosylation and sialylation in neonatal cord blood compared with maternal third-trimester serum. Beyond IgG, we identified a novel positive correlation between SES-region IgG sialylation and galactosylation and gestational age. Since IgA is not supposed to be transported across the placenta, these patterns likely reflect endogenous fetal IgA production and maturation, where longer gestation may allow for increased synthesis or more complex glycan processing. Notably, we were only able to quantify SES-region IgA modifications in cord blood, likely due to the low concentration of neonatal IgA. Lastly, while we observed initial associations between glycosylation and fetal sex or birthweight, these findings remain exploratory and should be investigated further in larger studies.

### Strengths and Limitations

A major strength of this study is that, to our knowledge, it is the first to investigate glycosylation profiles during the first trimester of pregnancy (9, 11 and 13 weeks GA), providing novel insights into early gestational immune modulation. Additionally, we uniquely compare IgG and IgA subtypes across different glycosylation regions, offering a more comprehensive view of antibody glycosylation. Our well-characterized periconceptional cohort, which includes detailed clinical parameters such as mode of conception and pregnancy outcomes, further enhances the clinical relevance of our findings. By adjusting for relevant covariates such as age and/or BMI, we aimed to estimate the independent associations between maternal characteristics and glycosylation changes. However, our study also has limitations. Maternal samples were collected at a maximum of 30 weeks gestation, while cord blood was measured at birth and only in a subset of participants, limiting direct longitudinal comparisons. Furthermore, the statistical power for subgroup analyses is limited. Another important limitation is the issue of multiple testing, given the large number of glycosylation traits and subgroup analyses performed. As a result, some associations may still reflect chance findings. However, we observed consistent and biologically plausible differences across subgroups such as women pregnant using ART and nulliparous women, and ours is currently the largest pregnancy cohort to examine these associations.

### Future Perspectives

The periconception period is a critical window, and the observed early glycosylation shifts may hold promise as predictive biomarkers. Evidence from other fields supports this potential: in rheumatoid arthritis, reduced galactosylation and sialylation of IgG Fc glycans precede disease onset and correlate with activity and treatment response [42]. Similarly, in metabolic and cardiovascular disease, lower IgG galactosylation has been linked to insulin resistance, hypertension, and increased cardiovascular risk [43]. Future studies could focus on associations of first trimester glycosylation changes and adverse pregnancy outcomes, and on first trimester placentation [9]. As we observed associations of pregnancy characteristics on glycosylation traits, mechanistic studies should account for the influence of these characteristics, including hormonal regulation, mode of conception, parity, and BMI. Lastly, the correlations observed for neonatal cord blood in a relatively small sample size warrant further investigation, ideally including matched maternal samples at the time of delivery to enable more accurate comparisons.

## Conclusion

IgG and IgA glycosylation traits are generally correlated and shift toward a more anti-inflammatory profile over the course of the first trimester. Maternal BMI, mode of conception, and nulliparity significantly influence glycosylation changes, highlighting the dynamic and adaptable nature of immunoglobulin glycosylation during pregnancy.

## Supplementary Information

Below is the link to the electronic supplementary material.

Supplementary file 1 (DOCX 63 KB)

## Data Availability

The datasets generated and/or analysed during the current study are not publicly available but are available from the corresponding author on reasonable request.

## Abbreviations

ADCC: Antibody-dependent cellular cytotoxicity
ART: Assisted reproductive technologies
BMI: Body mass index
CRL: Crown rump length
Fc: Fragment crystallizable region
FcγR: Fc gamma receptor
GA: Gestational age
Gal: Galactose
GlcNAc: N-Acetylglucosamine
IgA: Immunoglobulin A
IgG: Immunoglobulin G
IVF: In vitro fertilization
ICSI: Intracytoplasmic sperm injection
LMP: Last menstrual period
