# Supplementary material for: Longitudinal Profiling of IgG and IgA Glycosylation in Pregnancy Reveals Early Associations with BMI, Nulliparity and Conception Mode: The Rotterdam Periconception Cohort
Source: Reprod Sci. 2026 Sep 17;33(10):2081–93. doi: 10.1007/s43032-026-02190-9 (PMC13645990; doi:10.1007/s43032-026-02190-9)
Supplement: Supplementary file 1 [file 43032_2026_2190_MOESM1_ESM.docx]

**Supplementary Material**

Including:

- Supplementary Figures S1-S9
- Supplementary Results and Interpretation
- Supplementary Tables

**Supplementary Figures**

**Supplementary Figure S1.** Correlation matrix of glycosylation traits measured at 11 weeks gestational age.

**Supplementary Figure S2.** Correlation matrix of glycosylation traits measured at 22 weeks gestational age.

**Supplementary Figure S3.** Correlation matrix of glycosylation traits measured at 32 weeks gestational age.

**Supplementary Figure S4.** Relative abundance of glycosylation traits over pregnancy.

**Supplementary Figure S5.** Predicted gestational trajectories of IgG and IgA glycosylation traits by BMI level. Trajectories are derived from linear mixed-effects models with BMI as a continuous variable, showing predicted Z-scores across gestation for three representative BMI values: 18.5 kg/m² (normal weight), 25 kg/m² (overweight threshold), and 30 kg/m² (obese threshold). Each panel represents one glycosylation trait.

**Supplementary Figure S6 –** Predicted gestational trajectories of IgG and IgA glycosylation traits by parity. Trajectories are derived from linear mixed-effects models, showing predicted Z-scores across gestation for nulliparous women and parous women. Each panel represents one glycosylation trait.

**Supplementary Figure S7 –** Predicted gestational trajectories of IgG and IgA glycosylation traits by mode of conception. Trajectories are derived from linear mixed-effects models, showing predicted Z-scores across gestation for spontaneously conceived pregnancies and pregnancies achieved through assisted reproductive technology. Each panel represents one glycosylation trait. ART, assisted reproductive technology; BMI, body mass index.

B

A

**Supplementary Figure S8.** Significant differences in glycosylation trajectories for spontaneous pregnancies (A) and IVF/ICSI pregnancies (B) between nulliparous and multiparous women.

**Supplementary Figure S9**. Correlation matrix of measured glycosylation traits in neonatal cord blood with maternal and neonatal characteristics.

**Supplementary Results and Interpretation**

**Results of Correlation Analyses**

*Patterns of Bisected Glycans*

Bisected glycans were found to be positively correlated across different immunoglobulins and glycosylation sites, but tended to be negatively correlated with other modifications like sialylation and galactosylation. Specifically, strong positive correlations (r between 0.72 and 0.94) were found for the bisection levels of the four IgA N-glycosylation sites LAGY, LAGC, LSL, and TPL. Bisection of these four IgA N-glycosylation sites also showed moderate-to-strong correlations with IgG Fc N-glycosylation for the various subclasses (r 0.57 to 0.70).

Bisection showed predominantly negative correlations with sialylation and galactosylation. The strength of this inverse relationship varied, with the strongest negative correlations (r=−0.6 to −0.7) found between bisected LAGC, LAGY, and LSL glycans and the sialylation and galactosylation of LSL. Bisected IgG glycans displayed weaker negative correlations (r≈−0.3) with galactosylation and sialylation of LSL (IgA). Interestingly, for IgG1 bisection showed weak negative correlation with galactosylation (r=–0.17) and sialylation (r=–0.26), while such a negative correlation was not apparent for IgG4.

*Hierarchical Correlations in Galactosylation and Sialylation*

For terminal modifications like galactosylation and sialylation, the correlations followed a logical hierarchy. The strongest correlations were found within antibody classes, while correlations between IgG and IgA were weaker and less consistent.

Within the IgG class, IgG1 and IgG2 galactosylation were strongly correlated (r=0.73), while the correlations of IgG1 and IgG2 galactosylation with IgG4 galactosylation were more moderate (r=0.51 and 0.46, respectively). Similarly, IgG1 and IgG2 sialylation showed strong correlation (r=0.91). Again, sialylation of IgG1 and IgG2 showed moderate correlation with IgG4 sialylation (r=0.53 and 0.48, respectively).

Between antibody classes, correlations were less consistent. For instance, IgG1 and IgG2 galactosylation was positively correlated with LSL galactosylation (r=0.55 and 0.64, respectively). Similarly, for IgG1 and IgG2 sialylation, correlations with LSL sialylation were observed.(r=0.62 and 0.48, respectively). Of note, these glycosylation sites on IgG and IgA are structurally homologous, implying that the structural similarity of the glycosylation sites may play a role in steering galactosylation in a conserved way across antibody classes. In contrast, IgG1 Fc galactosylation showed only low (r=0.23) or no (r=0.02) correlation with LAGC and LAGY galactosylation, respectively. Interestingly, LAGC and LAGY glycopeptides which only differ in the absence or presence of the terminal tyrosine, showed virtually no correlation with respect to galactosylation levels (0.09) while their sialylation levels showed moderate correlation (r=0.37).

*Correlations in Fucosylation*

Core fucosylation on IgG1, IgG2, ENI, and TPL was moderately correlated (r=0.35 to 0.6), while fucosylation at other sites, such as SES and LAGC, seemed to be independently regulated. Of note, LAGC and LAGY fucosylation showed no correlation.

*IgA2 N-glycosylation*

The TPL and SES glycosylation sites of IgA2 are not shared with IgA1, and therefore provide insights into IgA2-specific N-glycosylation. TPL glycosylation traits correlated with various other immunoglobulin N-glycosylation traits. Interestingly, TPL fucosylation showed the strongest correlation with ENI fucosylation (r=0.52), followed by LAGY fucosylation (r=41) whilst there was hardly any correlation with LAGC fucosylation (r=0.12). These data point towards shared fucosylation programs of dimeric IgA2 as reflected in joining chain glycosylation (ENI) and glycosylation of the tyrosine-terminating LAGY glycopeptide. In contrast, SES glycosylation showed no strong correlations with other glycosylation sites, possibly reflecting the distant location of the SES glycosylation site in the CH1 domain of the IgA2 heavy chain.

*IgA1 hinge region O-glycosylation*

The O-glycosylation traits of the HYT hinge-region O-glycopeptide of IgA1 largely showed correlation with other traits of the HYT cluster, whilst no strong correlations with N-glycosylation features were observed.

**Comparison to Current Understanding in the Field**

Our findings regarding the correlations of IgG and IgA glycosylation traits align with previous studies conducted outside of pregnancy (Visconti 2024, Momcilovic 2020) Consistent with Visconti et al. and Momcilovic et al., we observed the strongest correlations between IgG subtypes and the IgA LSL, LAGY, LAGC, and TPL regions, particularly for N-glycan traits such as galactosylation, sialylation, and bisection (Visconti 2024, Momcilovic 2020). Glycosylation traits within the HYT region, reflecting O-glycosylation, did not correlate with other IgA or IgG features, suggesting a distinct regulatory mechanism. Visconti *et al.* attributed these correlations primarily to shared genetic factors, particularly through enzymes like MGAT3 and ST6GAL1 (Momcilovic 2020). In addition, the observed correlations may also reflect functional and structural parallels between immunoglobulin classes. In IgG molecules, the conserved N-glycosylation site at asparagine 297 (N297) is located on the heavy chain's Fc region, and is important for maintaining structural integrity and modulating effector functions, such as binding to Fc gamma receptors and complement activation (Dekkers 2017). Similarly, the N-glycosylation sites LSL, LAGC, LAGY, and TPL regions are also located on the heavy chain Fc region of IgA molecules (Woof 2011, Momcilovic 2020). What our study uniquely contributes is the demonstration that these correlation patterns are preserved during pregnancy. Moreover, despite changes in individual glycosylation traits over the course of pregnancy, the underlying correlation structure between traits was preserved across all trimesters, pointing towards preserved regulatory mechanisms.

**Supplementary Tables**

**Supplementary Table 1. Derived glycosylation traits with corresponding formulas.**

| **trait** | **formula** |
| --- | --- |
| IgGI_fucosylation | (1 * (IgGI1H3N4F1 + IgGI1H4N4F1 + IgGI1H5N4F1 + IgGI1H4N4F1S1 + IgGI1H5N4F1S1 + IgGI1H5N4F1S2 + IgGI1H3N5F1 + IgGI1H4N5F1 + IgGI1H5N5F1 + IgGI1H4N5F1S1 + IgGI1H5N5F1S1 + IgGI1H4N3F1)) / (IgGI1H3N4F1 + IgGI1H4N4F1 + IgGI1H5N4F1 + IgGI1H4N4F1S1 + IgGI1H5N4F1S1 + IgGI1H5N4F1S2 + IgGI1H3N5F1 + IgGI1H4N5F1 + IgGI1H5N5F1 + IgGI1H4N5F1S1 + IgGI1H5N5F1S1 + IgGI1H3N4 + IgGI1H4N4 + IgGI1H5N4 + IgGI1H5N4S1 + IgGI1H3N5 + IgGI1H4N5 + IgGI1H5N5 + IgGI1H5N5S1 + IgGI1H4N3F1) * 100 |
| IgGI_bisection | (1 * (IgGI1H3N5F1 + IgGI1H4N5F1 + IgGI1H5N5F1 + IgGI1H4N5F1S1 + IgGI1H5N5F1S1 + IgGI1H3N5 + IgGI1H4N5 + IgGI1H5N5 + IgGI1H5N5S1)) / (IgGI1H3N4F1 + IgGI1H4N4F1 + IgGI1H5N4F1 + IgGI1H4N4F1S1 + IgGI1H5N4F1S1 + IgGI1H5N4F1S2 + IgGI1H3N5F1 + IgGI1H4N5F1 + IgGI1H5N5F1 + IgGI1H4N5F1S1 + IgGI1H5N5F1S1 + IgGI1H3N4 + IgGI1H4N4 + IgGI1H5N4 + IgGI1H5N4S1 + IgGI1H3N5 + IgGI1H4N5 + IgGI1H5N5 + IgGI1H5N5S1 + IgGI1H4N3F1) * 100 |
| IgGI_galactosylation | (0.5 * (IgGI1H4N4F1 + IgGI1H4N4F1S1 + IgGI1H4N5F1 + IgGI1H4N5F1S1 + IgGI1H4N4 + IgGI1H4N5) + 1 * (IgGI1H5N4F1 + IgGI1H5N4F1S1 + IgGI1H5N4F1S2 + IgGI1H5N5F1 + IgGI1H5N5F1S1 + IgGI1H5N4 + IgGI1H5N4S1 + IgGI1H5N5 + IgGI1H5N5S1 + IgGI1H4N3F1)) / (IgGI1H3N4F1 + IgGI1H4N4F1 + IgGI1H5N4F1 + IgGI1H4N4F1S1 + IgGI1H5N4F1S1 + IgGI1H5N4F1S2 + IgGI1H3N5F1 + IgGI1H4N5F1 + IgGI1H5N5F1 + IgGI1H4N5F1S1 + IgGI1H5N5F1S1 + IgGI1H3N4 + IgGI1H4N4 + IgGI1H5N4 + IgGI1H5N4S1 + IgGI1H3N5 + IgGI1H4N5 + IgGI1H5N5 + IgGI1H5N5S1 + IgGI1H4N3F1) * 100 |
| IgGI_sialylation | (0.5 * (IgGI1H4N4F1S1 + IgGI1H5N4F1S1 + IgGI1H4N5F1S1 + IgGI1H5N5F1S1 + IgGI1H5N4S1 + IgGI1H5N5S1) + 1 * IgGI1H5N4F1S2) / (IgGI1H3N4F1 + IgGI1H4N4F1 + IgGI1H5N4F1 + IgGI1H4N4F1S1 + IgGI1H5N4F1S1 + IgGI1H5N4F1S2 + IgGI1H3N5F1 + IgGI1H4N5F1 + IgGI1H5N5F1 + IgGI1H4N5F1S1 + IgGI1H5N5F1S1 + IgGI1H3N4 + IgGI1H4N4 + IgGI1H5N4 + IgGI1H5N4S1 + IgGI1H3N5 + IgGI1H4N5 + IgGI1H5N5 + IgGI1H5N5S1 + IgGI1H4N3F1) * 100 |
| IgGI_hybrid | 1 * (IgGI1H6N3F1S1 + IgGI1H6N4F1) |
| IgGII_fucosylation | 1 * (IgGII1H3N4F1 + IgGII1H4N4F1 + IgGII1H5N4F1 + IgGII1H4N4F1S1 + IgGII1H5N4F1S1 + IgGII1H3N5F1 + IgGII1H4N5F1 + IgGII1H5N5F1 + IgGII1H5N5F1S1) |
| IgGII_bisection | 1 * (IgGII1H3N5F1 + IgGII1H4N5F1 + IgGII1H5N5F1 + IgGII1H5N5F1S1) |
| IgGII_galactosylation | 0.5 * (IgGII1H4N4F1 + IgGII1H4N4F1S1 + IgGII1H4N5F1 + IgGII1H4N4) + 1 * (IgGII1H5N4F1 + IgGII1H5N4F1S1 + IgGII1H5N5F1 + IgGII1H5N5F1S1) |
| IgGII_sialylation | 0.5 * (IgGII1H4N4F1S1 + IgGII1H5N4F1S1 + IgGII1H5N5F1S1) |
| IgGIV_bisection | 1 * (IgGIV1H3N5F1 + IgGIV1H4N5F1 + IgGIV1H5N5F1) |
| IgGIV_galactosylation | 0.5 * (IgGIV1H4N4F1 + IgGIV1H4N4F1S1 + IgGIV1H4N5F1) + 1 * (IgGIV1H5N4F1 + IgGIV1H5N4F1S1 + IgGIV1H5N5F1) |
| IgGIV_sialylation | 0.5 * (IgGIV1H4N4F1S1 + IgGIV1H5N4F1S1) |
| IgGI_sialylation_per_galactose | IgGI_sialylation / IgGI_galactosylation * 100 |
| IgGII_sialylation_per_galactose | IgGII_sialylation / IgGII_galactosylation * 100 |
| IgGIV_sialylation_per_galactose | IgGIV_sialylation / IgGIV_galactosylation * 100 |
| SES_fucosylation | 1 * (SES1H4N5F1 + SES1H4N5F1S1 + SES1H4N5F2 + SES1H4N5F2S1 + SES1H5N4F1S2 + SES1H5N5F1S1 + SES1H5N5F1S2 + SES1H5N6F1 + SES1H5N6F2 + SES1H5N6F1S1 + SES1H5N6F1S2 + SES1H5N6F2S2) |
| SES_antennary_fucosylation | 1 * (SES1H4N5F2 + SES1H4N5F2S1 + SES1H5N6F2 + SES1H5N6F2S2) |
| SES_bisection | 1 * (SES1H4N5F1 + SES1H4N5F1S1 + SES1H4N5F2 + SES1H4N5F2S1 + SES1H5N5S1 + SES1H5N5F1S1 + SES1H5N5F1S2 + SES1H5N6F1 + SES1H5N6F2 + SES1H5N6F1S1 + SES1H5N6F1S2 + SES1H5N6F2S2) |
| SES_galactosylation | 0.5 * (SES1H4N5F1 + SES1H4N5F1S1 + SES1H4N5F2 + SES1H4N5F2S1) + 1 * (SES1H5N4F1S2 + SES1H5N5S1 + SES1H5N5F1S1 + SES1H5N5F1S2) + 0.667 * (SES1H5N6F1 + SES1H5N6F2 + SES1H5N6F1S1 + SES1H5N6F1S2 + SES1H5N6F2S2) |
| SES_sialylation | 0.5 * (SES1H4N5F1S1 + SES1H4N5F2S1 + SES1H5N5S1 + SES1H5N5F1S1) + 1 * (SES1H5N4F1S2 + SES1H5N5F1S2) + 0.333 * SES1H5N6F1S1 + 0.667 * (SES1H5N6F1S2 + SES1H5N6F2S2) |
| SES_tri_antennary | 1 * (SES1H5N6F1 + SES1H5N6F2 + SES1H5N6F1S1 + SES1H5N6F1S2 + SES1H5N6F2S2) |
| SES_sialylation_per_galactose | SES_sialylation / SES_galactosylation * 100 |
| LSL_bisection | 1 * (LSL1H3N5 + LSL1H4N5 + LSL1H4N5S1 + LSL1H5N5S1) |
| LSL_galactosylation | 0.5 * (LSL1H4N5 + LSL1H4N5S1) + 1 * (LSL1H5N4S1 + LSL1H5N4S2 + LSL1H5N5S1) |
| LSL_sialylation | 0.5 * (LSL1H4N5S1 + LSL1H5N4S1 + LSL1H5N5S1) + 1 * LSL1H5N4S2 |
| LSL_sialylation_per_galactose | LSL_sialylation / LSL_galactosylation * 100 |
| TPL_fucosylation | 1 * (TPL1H4N5F1S1 + TPL1H5N4F1S1 + TPL1H5N4F1S2 + TPL1H5N5F1S1 + TPL1H5N5F1S2 + TPL1H6N5F1S1) |
| TPL_bisection | 1 * (TPL1H4N5F1S1 + TPL1H5N5S1 + TPL1H5N5F1S1 + TPL1H5N5F1S2) |
| TPL_galactosylation | 0.5 * TPL1H4N5F1S1 + 1 * (TPL1H5N4F1S1 + TPL1H5N4F1S2 + TPL1H5N5S1 + TPL1H5N5F1S1 + TPL1H5N5F1S2 + TPL1H6N5F1S1) |
| TPL_sialylation | 0.5 * (TPL1H4N5F1S1 + TPL1H5N4F1S1 + TPL1H5N5S1 + TPL1H5N5F1S1) + 1 * (TPL1H5N4F1S2 + TPL1H5N5F1S2) + 0.333 * TPL1H6N5F1S1 |
| TPL_sialylation_per_galactose | TPL_sialylation / TPL_galactosylation * 100 |
| HYT_sialic_acids | (2 * (HYT1H2N3S2 + HYT1H2N4S2 + HYT1H3N3S2 + HYT1H3N4S2 + HYT1H3N5S2 + HYT1H3N6S2 + HYT1H4N4S2 + HYT1H4N5S2 + HYT1H4N6S2 + HYT1H5N5S2 + HYT1H5N6S2) + 1 * (HYT1H2N5S1 + HYT1H3N3S1 + HYT1H3N4S1 + HYT1H3N5S1 + HYT1H3N6S1 + HYT1H4N4S1 + HYT1H4N5S1 + HYT1H5N5S1) + 3 * (HYT1H3N3S3 + HYT1H3N4S3 + HYT1H3N5S3 + HYT1H3N6S3 + HYT1H4N4S3 + HYT1H4N5S3 + HYT1H4N6S3 + HYT1H5N5S3 + HYT1H5N6S3) + 4 * (HYT1H3N3S4 + HYT1H3N4S4 + HYT1H4N4S4 + HYT1H4N5S4 + HYT1H5N5S4 + HYT1H3N5S4) + 5 * HYT1H4N4S5) / 100 |
| HYT_galactoses | (2 * (HYT1H2N3S2 + HYT1H2N4S2 + HYT1H2N5S1) + 3 * (HYT1H3N3S1 + HYT1H3N3S2 + HYT1H3N3S3 + HYT1H3N3S4 + HYT1H3N4 + HYT1H3N4S1 + HYT1H3N4S2 + HYT1H3N4S3 + HYT1H3N4S4 + HYT1H3N5 + HYT1H3N5S1 + HYT1H3N5S2 + HYT1H3N5S3 + HYT1H3N6S1 + HYT1H3N6S2 + HYT1H3N6S3 + HYT1H3N5S4) + 4 * (HYT1H4N4 + HYT1H4N4S1 + HYT1H4N4S2 + HYT1H4N4S3 + HYT1H4N4S4 + HYT1H4N4S5 + HYT1H4N5 + HYT1H4N5S1 + HYT1H4N5S2 + HYT1H4N5S3 + HYT1H4N5S4 + HYT1H4N6S2 + HYT1H4N6S3 + HYT1H4N6) + 5 * (HYT1H5N5 + HYT1H5N5S1 + HYT1H5N5S2 + HYT1H5N5S3 + HYT1H5N5S4 + HYT1H5N6S3 + HYT1H5N6S2)) / 100 |
| HYT_galnacs | (3 * (HYT1H2N3S2 + HYT1H3N3S1 + HYT1H3N3S2 + HYT1H3N3S3 + HYT1H3N3S4) + 4 * (HYT1H2N4S2 + HYT1H3N4 + HYT1H3N4S1 + HYT1H3N4S2 + HYT1H3N4S3 + HYT1H3N4S4 + HYT1H4N4 + HYT1H4N4S1 + HYT1H4N4S2 + HYT1H4N4S3 + HYT1H4N4S4 + HYT1H4N4S5) + 5 * (HYT1H2N5S1 + HYT1H3N5 + HYT1H3N5S1 + HYT1H3N5S2 + HYT1H3N5S3 + HYT1H4N5 + HYT1H4N5S1 + HYT1H4N5S2 + HYT1H4N5S3 + HYT1H4N5S4 + HYT1H5N5 + HYT1H5N5S1 + HYT1H5N5S2 + HYT1H5N5S3 + HYT1H5N5S4 + HYT1H3N5S4) + 6 * (HYT1H3N6S1 + HYT1H3N6S2 + HYT1H3N6S3 + HYT1H4N6S2 + HYT1H4N6S3 + HYT1H5N6S3 + HYT1H4N6 + HYT1H5N6S2)) / 100 |
| HYT_Tn_antigens | (1 * (HYT1H2N3S2 + HYT1H3N4 + HYT1H3N4S1 + HYT1H3N4S2 + HYT1H3N4S3 + HYT1H3N4S4 + HYT1H4N5 + HYT1H4N5S1 + HYT1H4N5S2 + HYT1H4N5S3 + HYT1H4N5S4 + HYT1H5N6S3 + HYT1H5N6S2) + 2 * (HYT1H2N4S2 + HYT1H3N5 + HYT1H3N5S1 + HYT1H3N5S2 + HYT1H3N5S3 + HYT1H4N6S2 + HYT1H4N6S3 + HYT1H4N6 + HYT1H3N5S4) + 3 * (HYT1H2N5S1 + HYT1H3N6S1 + HYT1H3N6S2 + HYT1H3N6S3)) / 100 |
| HYT_T_antigens | (1 * (HYT1H2N5S1 + HYT1H3N3S2 + HYT1H3N4S2 + HYT1H3N5S2 + HYT1H3N6S2 + HYT1H4N4S3 + HYT1H4N5S3 + HYT1H4N6S3 + HYT1H5N5S4) + 2 * (HYT1H3N3S1 + HYT1H3N4S1 + HYT1H3N5S1 + HYT1H3N6S1 + HYT1H4N4S2 + HYT1H4N5S2 + HYT1H4N6S2 + HYT1H5N5S3 + HYT1H5N6S3) + 3 * (HYT1H3N4 + HYT1H3N5 + HYT1H4N4S1 + HYT1H4N5S1 + HYT1H5N5S2 + HYT1H5N6S2) + 4 * (HYT1H4N4 + HYT1H4N5 + HYT1H5N5S1 + HYT1H4N6) + 5 * HYT1H5N5) / 100 |
| HYT_sT_antigens | (2 * (HYT1H2N3S2 + HYT1H2N4S2 + HYT1H3N3S2 + HYT1H3N4S2 + HYT1H3N5S2 + HYT1H3N6S2 + HYT1H4N4S2 + HYT1H4N5S2 + HYT1H4N6S2 + HYT1H5N5S2 + HYT1H5N6S2) + 1 * (HYT1H2N5S1 + HYT1H3N3S1 + HYT1H3N4S1 + HYT1H3N5S1 + HYT1H3N6S1 + HYT1H4N4S1 + HYT1H4N5S1 + HYT1H5N5S1) + 3 * (HYT1H3N3S3 + HYT1H3N3S4 + HYT1H3N4S3 + HYT1H3N4S4 + HYT1H3N5S3 + HYT1H3N6S3 + HYT1H4N4S3 + HYT1H4N5S3 + HYT1H4N6S3 + HYT1H5N5S3 + HYT1H5N6S3 + HYT1H3N5S4) + 4 * (HYT1H4N4S4 + HYT1H4N4S5 + HYT1H4N5S4 + HYT1H5N5S4)) / 100 |
| HYT_disialylated_O_antigens | 1 * (HYT1H3N3S4 + HYT1H3N4S4 + HYT1H4N4S5 + HYT1H3N5S4) |
| HYT_sialic_acids_per_galactose | HYT_sialic_acids / HYT_galactoses |
| HYT_galactoses_per_galnac | HYT_galactoses / HYT_galnacs |
| ENI_fucosylation | (1 * (ENI1H5N4F1S1 + ENI1H5N4F1S2)) / (ENI1H5N4F1S1 + ENI1H5N4F1S2 + ENI1H5N4S1 + ENI1H5N4S2 + ENI1H5N5S1) * 100 |
| ENI_sialylation | (0.5 * (ENI1H5N4F1S1 + ENI1H5N4S1 + ENI1H5N5S1) + 1 * (ENI1H5N4F1S2 + ENI1H5N4S2)) / (ENI1H5N4F1S1 + ENI1H5N4F1S2 + ENI1H5N4S1 + ENI1H5N4S2 + ENI1H5N5S1) * 100 |
| ENI_hybrid | 1 * (ENI1H5N3F1S1 + ENI1H5N3S1) |
| LAGC_fucosylation | 1 * (LAGC1H5N4F1S1 + LAGC1H5N4F1S2 + LAGC1H5N5F1S1 + LAGC1H5N5F1S2 + LAGC1H6N5F1S1 + LAGC1H6N5F1S2 + LAGC1H6N5F1S3 + LAGC1H6N6F1S2 + LAGC1H5N4F1S2Su1 + LAGC1H6N5F1S1Su1) |
| LAGC_bisection | 1 * (LAGC1H5N5F1S1 + LAGC1H5N5F1S2 + LAGC1H5N6S2 + LAGC1H6N6F1S2) |
| LAGC_galactosylation | 1 * (LAGC1H5N4F1S1 + LAGC1H5N4F1S2 + LAGC1H5N5F1S1 + LAGC1H5N5F1S2 + LAGC1H6N5F1S1 + LAGC1H6N5F1S2 + LAGC1H6N5F1S3 + LAGC1H6N6F1S2 + LAGC1H5N4F1S2Su1 + LAGC1H6N5F1S1Su1) + 0.667 * LAGC1H5N6S2 |
| LAGC_sialylation | 0.5 * (LAGC1H5N4F1S1 + LAGC1H5N5F1S1) + 1 * (LAGC1H5N4F1S2 + LAGC1H5N5F1S2 + LAGC1H6N5F1S3 + LAGC1H5N4F1S2Su1) + 0.333 * (LAGC1H6N5F1S1 + LAGC1H6N5F1S1Su1) + 0.667 * (LAGC1H6N5F1S2 + LAGC1H5N6S2 + LAGC1H6N6F1S2) |
| LAGC_tri_antennary | 1 * (LAGC1H6N5F1S1 + LAGC1H6N5F1S2 + LAGC1H6N5F1S3 + LAGC1H5N6S2 + LAGC1H6N6F1S2 + LAGC1H6N5F1S1Su1) |
| LAGY_fucosylation | (1 * (LAGY1H4N5F1 + LAGY1H5N4F1S1 + LAGY1H5N4F1S2 + LAGY1H5N5F1 + LAGY1H5N5F1S1 + LAGY1H5N5F1S2 + LAGY1H6N5F1S1 + LAGY1H6N5F1S2 + LAGY1H5N4F1S2Su1 + LAGY1H5N5F1S2Su1 + LAGY1H6N5F1S2Su1 + LAGY1H6N6F1S2Su1)) / (LAGY1H4N5F1 + LAGY1H5N4S1 + LAGY1H5N4S2 + LAGY1H5N4F1S1 + LAGY1H5N4F1S2 + LAGY1H5N5F1 + LAGY1H5N5F1S1 + LAGY1H5N5F1S2 + LAGY1H6N5F1S1 + LAGY1H6N5F1S2 + LAGY1H5N4F1S2Su1 + LAGY1H5N5F1S2Su1 + LAGY1H6N5F1S2Su1 + LAGY1H6N6F1S2Su1) * 100 |
| LAGY_bisection | (1 * (LAGY1H4N5F1 + LAGY1H5N5F1 + LAGY1H5N5F1S1 + LAGY1H5N5F1S2 + LAGY1H5N5F1S2Su1 + LAGY1H6N6F1S2Su1)) / (LAGY1H4N5F1 + LAGY1H5N4S1 + LAGY1H5N4S2 + LAGY1H5N4F1S1 + LAGY1H5N4F1S2 + LAGY1H5N5F1 + LAGY1H5N5F1S1 + LAGY1H5N5F1S2 + LAGY1H6N5F1S1 + LAGY1H6N5F1S2 + LAGY1H5N4F1S2Su1 + LAGY1H5N5F1S2Su1 + LAGY1H6N5F1S2Su1 + LAGY1H6N6F1S2Su1) * 100 |
| LAGY_galactosylation | (0.5 * LAGY1H4N5F1 + 1 * (LAGY1H5N4S1 + LAGY1H5N4S2 + LAGY1H5N4F1S1 + LAGY1H5N4F1S2 + LAGY1H5N5F1 + LAGY1H5N5F1S1 + LAGY1H5N5F1S2 + LAGY1H6N5F1S1 + LAGY1H6N5F1S2 + LAGY1H5N4F1S2Su1 + LAGY1H5N5F1S2Su1 + LAGY1H6N5F1S2Su1 + LAGY1H6N6F1S2Su1)) / (LAGY1H4N5F1 + LAGY1H5N4S1 + LAGY1H5N4S2 + LAGY1H5N4F1S1 + LAGY1H5N4F1S2 + LAGY1H5N5F1 + LAGY1H5N5F1S1 + LAGY1H5N5F1S2 + LAGY1H6N5F1S1 + LAGY1H6N5F1S2 + LAGY1H5N4F1S2Su1 + LAGY1H5N5F1S2Su1 + LAGY1H6N5F1S2Su1 + LAGY1H6N6F1S2Su1) * 100 |
| LAGY_sialylation | (0.5 * (LAGY1H5N4S1 + LAGY1H5N4F1S1 + LAGY1H5N5F1S1) + 1 * (LAGY1H5N4S2 + LAGY1H5N4F1S2 + LAGY1H5N5F1S2 + LAGY1H5N4F1S2Su1 + LAGY1H5N5F1S2Su1) + 0.333 * LAGY1H6N5F1S1 + 0.667 * (LAGY1H6N5F1S2 + LAGY1H6N5F1S2Su1 + LAGY1H6N6F1S2Su1)) / (LAGY1H4N5F1 + LAGY1H5N4S1 + LAGY1H5N4S2 + LAGY1H5N4F1S1 + LAGY1H5N4F1S2 + LAGY1H5N5F1 + LAGY1H5N5F1S1 + LAGY1H5N5F1S2 + LAGY1H6N5F1S1 + LAGY1H6N5F1S2 + LAGY1H5N4F1S2Su1 + LAGY1H5N5F1S2Su1 + LAGY1H6N5F1S2Su1 + LAGY1H6N6F1S2Su1) * 100 |
| LAGY_tri_antennary | (1 * (LAGY1H6N5F1S1 + LAGY1H6N5F1S2 + LAGY1H6N5F1S2Su1 + LAGY1H6N6F1S2Su1)) / (LAGY1H4N5F1 + LAGY1H5N4S1 + LAGY1H5N4S2 + LAGY1H5N4F1S1 + LAGY1H5N4F1S2 + LAGY1H5N5F1 + LAGY1H5N5F1S1 + LAGY1H5N5F1S2 + LAGY1H6N5F1S1 + LAGY1H6N5F1S2 + LAGY1H5N4F1S2Su1 + LAGY1H5N5F1S2Su1 + LAGY1H6N5F1S2Su1 + LAGY1H6N6F1S2Su1) * 100 |
| LAGC_sialylation_per_galactose | LAGC_sialylation / LAGC_galactosylation * 100 |
| LAGY_sialylation_per_galactose | LAGY_sialylation / LAGY_galactosylation * 100 |

**Supplementary Table 2. Overview of IgA glycopeptide clusters, corresponding IgA subclasses, and glycosylation sites indicated by residue number according to the IgA1 or IgA2 heavy chain sequence (Bondt et al 2014, Dotz et al 2021).**

| **Cluster** | **IgA subtype** | **Glycosylation site** |
| --- | --- | --- |
| SES | IgA2 | N47 |
| LSL | IgA1/IgA2 | N144/N131 |
| TPL | IgA2 | N205 |
| LAGC | IgA1/IgA2 | N327/N340 |
| LAGY | IgA1/IgA2 | N327/N340 |
| HYT | IgA1 | T106/T109/S111/S113/T114/T117 (O-glycans) |
| ENI |  | Joining Chain N71 |

**Supplementary Table 3.** **Mean and Standard Deviation (SD) of Measured Glycosylation Traits Across Pregnancy**. The ‘Significant’ column indicates whether any of the predefined pairwise comparisons between time points were statistically significant (FDR-adjusted p < 0.05), based on linear mixed-effects models with a random intercept for each subject: (a) 11–22 weeks, (b) 11–32 weeks, (c) 22–32 weeks, (d) 9–11 weeks, and (e) 9–13 weeks.

| Variable | 9 | 11 | 13 | 22 | 32 | Significant |
| --- | --- | --- | --- | --- | --- | --- |
| N | 108 | 192 | 86 | 187 | 78 |  |
| IgGI_fucosylation | 91.62 (3.65) | 91.76 (3.45) | 92.28 (3.11) | 91.68 (3.48) | 91.60 (3.42) | a,b,c |
| IgGI_bisection | 15.88 (2.86) | 15.13 (2.58) | 14.79 (2.63) | 14.89 (2.51) | 14.93 (2.36) | a,d,e |
| IgGI_galactosylation | 66.02 (5.80) | 67.62 (4.95) | 68.21 (5.38) | 70.11 (5.14) | 70.45 (5.04) | a,b,c,d,e |
| IgGI_sialylation | 11.32 (1.84) | 11.94 (1.78) | 12.31 (1.83) | 12.74 (1.91) | 12.69 (1.91) | a,b,d,e |
| IgGI_hybrid | 0.26 (0.05) | 0.27 (0.06) | 0.27 (0.05) | 0.28 (0.06) | 0.28 (0.05) | a,b |
| IgGII_fucosylation | 99.27 (0.39) | 99.34 (0.35) | 99.41 (0.32) | 99.32 (0.36) | 99.31 (0.37) | b, c |
| IgGII_bisection | 12.09 (1.84) | 11.61 (1.94) | 11.37 (2.09) | 11.51 (1.89) | 11.54 (1.90) |  |
| IgGII_galactosylation | 60.71 (4.50) | 62.38 (4.99) | 63.56 (5.23) | 65.43 (4.96) | 65.68 (5.14) | a,b,c,d,e |
| IgGII_sialylation | 9.61 (1.55) | 10.28 (1.71) | 10.82 (1.93) | 11.28 (1.82) | 11.28 (1.86) | a,b,d,e |
| IgGIV_bisection | 12.44 (2.59) | 12.04 (2.87) | 11.70 (2.92) | 12.31 (3.02) | 12.61 (3.12) | a,b,c |
| IgGIV_galactosylation | 58.47 (6.24) | 58.97 (7.06) | 59.39 (7.29) | 61.43 (7.31) | 60.77 (7.06) | a,b,d,e |
| IgGIV_sialylation | 15.96 (2.64) | 16.40 (3.12) | 16.67 (3.27) | 17.65 (3.42) | 17.45 (3.33) | a,b,d,e |
| IgGI_sialylation_per_galactose | 17.09 (1.89) | 17.61 (1.86) | 18.01 (1.87) | 18.12 (1.93) | 17.95 (1.92) | a,b,e |
| IgGII_sialylation_per_galactose | 15.80 (1.98) | 16.43 (1.95) | 16.94 (2.12) | 17.18 (2.02) | 17.11 (1.97) | a,b,d,e |
| IgGIV_sialylation_per_galactose | 27.16 (2.29) | 27.60 (2.87) | 27.84 (3.36) | 28.50 (2.99) | 28.51 (2.87) | a,b |
| SES_fucosylation | 96.57 (1.00) | 96.51 (0.92) | 96.40 (0.94) | 96.97 (0.96) | 96.95 (0.89) | a,b |
| SES_antennary_fucosylation | 4.61 (1.27) | 4.34 (1.11) | 4.20 (1.12) | 4.27 (1.26) | 4.74 (1.59) | b,c,d,e |
| SES_bisection | 98.28 (1.32) | 98.26 (1.16) | 98.16 (1.19) | 98.22 (1.04) | 98.13 (1.33) | b |
| SES_galactosylation | 58.34 (1.73) | 58.46 (1.63) | 58.78 (1.58) | 59.59 (1.48) | 59.98 (1.88) | a,b,c,e |
| SES_sialylation | 50.72 (1.78) | 50.85 (1.79) | 50.94 (1.82) | 51.45 (1.85) | 52.04 (2.05) | a,b,c |
| SES_tri_antennary | 27.77 (4.27) | 28.38 (4.36) | 29.37 (4.14) | 35.90 (4.92) | 36.39 (5.21) | a,b,c,d,e |
| SES_sialylation_per_galactose | 86.94 (1.85) | 86.99 (1.86) | 86.66 (1.85) | 86.33 (2.07) | 86.76 (2.08) | a,c,e |
| LSL_bisection | 44.48 (5.80) | 43.49 (5.94) | 43.09 (5.89) | 46.79 (5.94) | 49.90 (6.16) | a,b,c |
| LSL_galactosylation | 85.37 (2.99) | 85.99 (3.03) | 86.26 (3.13) | 83.31 (3.72) | 80.76 (4.25) | a,b,c |
| LSL_sialylation | 48.65 (2.53) | 49.28 (2.65) | 49.59 (2.68) | 47.82 (2.92) | 46.02 (3.05) | a,b,c |
| LSL_sialylation_per_galactose | 56.97 (1.70) | 57.29 (1.66) | 57.47 (1.67) | 57.38 (1.69) | 56.95 (1.56) | b,c |
| TPL_fucosylation | 97.65 (0.61) | 97.69 (0.56) | 97.80 (0.54) | 97.58 (0.61) | 97.51 (0.60) | a,b,c |
| TPL_bisection | 50.83 (6.75) | 49.71 (7.38) | 48.68 (7.59) | 51.31 (7.08) | 52.93 (7.20) | a,b,c |
| TPL_galactosylation | 99.54 (0.18) | 99.56 (0.17) | 99.59 (0.16) | 99.45 (0.22) | 99.33 (0.27) | a,b,c |
| TPL_sialylation | 61.87 (1.92) | 62.12 (1.82) | 62.39 (2.04) | 62.12 (1.80) | 61.68 (1.79) | b,c,e |
| TPL_sialylation_per_galactose | 62.16 (1.91) | 62.39 (1.80) | 62.65 (2.03) | 62.47 (1.78) | 62.09 (1.78) | b,c,e |
| HYT_sialic_acids | 2.29 (0.23) | 2.28 (0.23) | 2.26 (0.21) | 2.21 (0.22) | 2.15 (0.21) | a,b,c |
| HYT_galactoses | 3.65 (0.08) | 3.66 (0.08) | 3.67 (0.07) | 3.67 (0.07) | 3.69 (0.08) | a,b,c |
| HYT_galnacs | 4.49 (0.04) | 4.49 (0.04) | 4.48 (0.04) | 4.49 (0.04) | 4.49 (0.04) | a,b |
| HYT_Tn_antigens | 0.83 (0.08) | 0.83 (0.08) | 0.82 (0.08) | 0.82 (0.08) | 0.80 (0.08) | b,c |
| HYT_T_antigens | 1.39 (0.21) | 1.42 (0.22) | 1.44 (0.20) | 1.49 (0.21) | 1.57 (0.20) | a,b,c |
| HYT_sT_antigens | 2.26 (0.22) | 2.25 (0.22) | 2.23 (0.20) | 2.19 (0.21) | 2.12 (0.20) | a,b,c |
| HYT_disialylated_O_antigens | 3.05 (0.88) | 2.96 (0.85) | 2.92 (0.77) | 2.93 (0.83) | 2.75 (0.77) | b,c |
| HYT_sialic_acids_per_galactose | 0.63 (0.06) | 0.62 (0.06) | 0.62 (0.06) | 0.60 (0.06) | 0.58 (0.05) | a,b,c |
| HYT_galactoses_per_galnac | 0.81 (0.02) | 0.82 (0.02) | 0.82 (0.02) | 0.82 (0.02) | 0.82 (0.02) | b,c |
| ENI_fucosylation | 23.84 (3.18) | 24.18 (3.24) | 24.43 (3.36) | 24.10 (3.08) | 24.41 (3.03) | c |
| ENI_sialylation | 84.22 (1.74) | 84.65 (1.92) | 84.87 (2.19) | 84.01 (1.90) | 83.67 (1.93) | a,b,c |
| ENI_hybrid | 22.08 (2.61) | 22.22 (2.65) | 22.69 (2.62) | 23.63 (2.65) | 24.64 (2.67) | a,b,c,e |
| LAGC_fucosylation | 98.02 (0.25) | 98.05 (0.17) | 98.04 (0.27) | 98.09 (0.18) | 98.07 (0.31) |  |
| LAGC_bisection | 42.22 (3.42) | 41.33 (3.71) | 40.99 (3.86) | 41.89 (3.56) | 42.97 (3.52) | a,b,c |
| LAGC_galactosylation | 99.34 (0.08) | 99.35 (0.06) | 99.35 (0.09) | 99.36 (0.06) | 99.36 (0.10) |  |
| LAGC_sialylation | 85.95 (1.57) | 86.34 (1.46) | 86.61 (1.58) | 86.06 (1.62) | 85.64 (1.60) | a,b,c,d,e |
| LAGC_tri_antennary | 18.96 (1.92) | 18.91 (1.86) | 18.84 (1.73) | 19.14 (2.09) | 19.35 (2.12) | a,b |
| LAGY_fucosylation | 95.52 (1.64) | 95.42 (1.57) | 95.36 (1.60) | 95.55 (1.55) | 95.53 (1.54) |  |
| LAGY_bisection | 50.39 (4.64) | 49.51 (4.75) | 49.13 (4.55) | 51.56 (4.78) | 53.39 (4.83) | a,b,c |
| LAGY_galactosylation | 99.16 (0.31) | 99.21 (0.30) | 99.27 (0.31) | 99.10 (0.35) | 98.90 (0.49) | a,b,c,e |
| LAGY_sialylation | 83.81 (2.65) | 84.29 (2.50) | 84.72 (2.44) | 84.23 (2.56) | 83.61 (2.72) | b,c,d,e |
| LAGY_tri_antennary | 4.54 (0.49) | 4.59 (0.51) | 4.63 (0.47) | 4.53 (0.46) | 4.40 (0.47) | b,c |
| LAGC_sialylation_per_galactose | 86.52 (1.58) | 86.90 (1.47) | 87.18 (1.61) | 86.61 (1.62) | 86.20 (1.62) | a,b,c,d,e |
| LAGY_sialylation_per_galactose | 84.52 (2.50) | 84.95 (2.33) | 85.34 (2.25) | 84.99 (2.37) | 84.53 (2.45) | b,c,d,e |

**Supplementary Table 4.** Glycosylation traits in mother-neonate pairs, percentage and 95% CI, in the first (T1), second (T2), and third trimester (T3) and in cord blood (CB). a = t-test between T3 and CB.

|  | **T1** | **T2** | **T3** | **CB** | **p-value^a^** | **p-value^a^** |
| --- | --- | --- | --- | --- | --- | --- |
| N | 73 | 69 | 72 | 78 |  | **FDR adj** |
| IgGI_fucosylation | 92.21 (3.17) | 92.20 (3.23) | 91.89 (3.40) | 91.38 (3.65) | 0,386 | 0,446 |
| IgGI_bisection | 14.95 (2.87) | 14.91 (2.89) | 14.73 (2.66) | 15.44 (2.81) | 0,117 | 0,161 |
| IgGI_galactosylation | 67.01 (5.14) | 69.25 (5.96) | 69.97 (5.37) | 71.82 (5.41) | **0,038** | 0,065 |
| IgGI_sialylation | 11.98 (1.75) | 12.71 (2.09) | 12.74 (1.96) | 13.44 (1.90) | **0,029** | 0,052 |
| IgGI_hybrid | 0.26 (0.05) | 0.27 (0.06) | 0.28 (0.05) | 0.26 (0.05) | **0,043** | 0,068 |
| IgGII_fucosylation | 99.33 (0.33) | 99.33 (0.35) | 99.30 (0.35) | 99.35 (0.44) | 0,464 | 0,510 |
| IgGII_bisection | 11.17 (2.08) | 11.10 (2.04) | 11.15 (2.08) | 11.30 (2.20) | 0,660 | 0,691 |
| IgGII_galactosylation | 61.78 (5.67) | 65.08 (5.53) | 65.13 (5.80) | 68.16 (6.28) | **0,003** | **0,006** |
| IgGII_sialylation | 10.34 (1.79) | 11.36 (1.86) | 11.34 (1.92) | 13.53 (2.19) | **<0,001** | **<0,001** |
| IgGIV_bisection | 11.77 (2.96) | 12.02 (3.17) | 12.32 (3.26) | 12.34 (3.45) | 0,974 | 0,974 |
| IgGIV_galactosylation | 58.04 (7.53) | 60.65 (8.10) | 59.76 (7.04) | 65.87 (7.06) | **<0,001** | **<0,001** |
| IgGIV_sialylation | 16.14 (3.23) | 17.36 (3.60) | 17.02 (3.04) | 19.13 (3.40) | **<0,001** | **<0,001** |
| IgGI_sialylation_per_galactose | 17.83 (1.80) | 18.28 (2.05) | 18.16 (1.95) | 18.67 (1.81) | 0,096 | 0,141 |
| IgGII_sialylation_per_galactose | 16.66 (1.84) | 17.40 (1.90) | 17.34 (1.90) | 19.81 (2.40) | **<0,001** | **<0,001** |
| IgGIV_sialylation_per_galactose | 27.54 (3.03) | 28.32 (3.18) | 28.31 (2.78) | 28.85 (2.90) | 0,300 | 0,388 |
| SES_fucosylation | 96.55 (0.96) | 96.85 (0.98) | 96.77 (0.97) | 96.07 (1.27) | **<0,001** | **<0,001** |
| SES_antennary_fucosylation | 4.39 (1.07) | 4.32 (1.20) | 4.93 (1.77) | 8.78 (2.11) | **<0,001** | **<0,001** |
| SES_bisection | 98.33 (1.10) | 98.35 (0.92) | 98.29 (1.35) | 99.75 (0.12) | **<0,001** | **<0,001** |
| SES_galactosylation | 58.26 (1.52) | 59.36 (1.44) | 59.74 (1.91) | 55.85 (1.02) | **<0,001** | **<0,001** |
| SES_sialylation | 50.47 (1.85) | 50.97 (1.92) | 51.62 (2.11) | 48.07 (1.19) | **<0,001** | **<0,001** |
| SES_tri_antennary | 28.34 (4.76) | 35.45 (4.68) | 36.14 (5.37) | 21.27 (4.54) | **<0,001** | **<0,001** |
| SES_sialylation_per_galactose | 86.61 (1.94) | 85.86 (2.14) | 86.42 (2.28) | 86.09 (1.93) | 0,367 | 0,446 |

**Supplementary Table 5.** Baseline characteristics, stratified by mode of conception.

|  | Spontaneous conception | ART | p-value |
| --- | --- | --- | --- |
| N | 103 | 83 |  |
|  |  |  |  |
| Patient characteristics |  |  |  |
| Maternal age, mean (SD) | 31.5 (4.4) | 32.8 (4.5) | **0.043** |
| Maternal BMI, median [IQR] | 25.5 [22.6, 29.9] | 24.1 [21.8, 27.7] | **0.034** |
| Country of origin, n (%) |  |  | 0.138 |
| Dutch | 84 (81.6) | 72 (86.7) |  |
| Western, not Dutch | 2 (1.9) | 3 (3.6) |  |
| Non-Western | 11 (10.7) | 8 (9.6) |  |
| Missing | 6 (5.0) | 0 (0.0) |  |
| Smoking periconceptional, n (%) | 13 (12.6) | 12 (14.5) | 0.115 |
| Missing | 8 (6.7) | 1 (1.2) |  |
| Pre-existing hypertension | 4 (3.9) | 1 (1.2) | 0.157 |
| Missing | 0 (0.0) | 2 (2.4) |  |
| Pre-existing diabetes mellitus |  |  |  |
| Type I | 2 (1.9) | 0 (0.0) | 0.575 |
| Type II | 0 (0.0) | 0 (0.0) | NA |
|  |  |  |  |
| Pregnancy characteristics |  |  |  |
| Mode of conception, n (%) |  |  | **<0.001** |
| Spontaneous | 103 (86.6) | 0 (0.0) |  |
| IVF | 0 (0.0) | 33 (39.8) |  |
| ICSI | 0 (0.0) | 50 (60.2) |  |
| Nulliparity, n (%) | 37 (35.9) | 64 (77.1) | **<0.001** |
| Missing | 0 (0.0) | 1 (1.2) |  |
| Gestational age at birth in days, median [IQR] | 272 [264, 279] | 274 [267, 278] | 0.277 |
| Neonatal birthweight percentile, median [IQR] | 51 [18, 72] | 44 [22, 71] | 0.924 |
| Pregnancy complication, n (%) |  |  |  |
| Preeclampsia | 3 (2.9) | 3 (3.6) | 0.273 |
| with onset <34 weeks | 1 (1.0) | 2 (2.4) | 0.332 |
| Pregnancy induced hypertension | 10 (9.7) | 4 (4.8) | 0.137 |
| Gestational diabetes | 14 (13.6) | 15 (18.1) | 0.189 |
| Missing | 0 (0.0) | 2 (2.4) |  |

**Supplementary Table 6.** Sensitivity analysis: significant main effects from linear mixed models comparing nulliparous and multiparous women, stratified by mode of conception.

| **Variable** | **Nulliparity (3)** | **Nulliparity (3), only spontaneous conception** | **Nulliparity (3), only IVF/ICSI pregnancy** |
| --- | --- | --- | --- |
| IgGI | X | Hybrid ↓ | X |
| IgGII | X | X | X |
| IgGIV | X | X | Bisection ↑ |
| LAGC | X | Bisection ↑ | X |
| LAGY | Galactosylation ↑ | X | Fucosylation ↑  Galactosylation ↑  Sialylation ↑  Sialylation per galactose ↑ |
| LSL | X | X | X |
| TPL | X | Bisection ↑ | X |
| SES | X | X | X |
| ENI | X | X | X |
| HYT | Galactoses ↓  Galactoses per GalNac ↓  Tn antigens ↑ | X | X |

(3) adjusted for maternal age and BMI
